# Potential Mechanism of Dermal Wound Treatment With Preparations From the Skin Gel of Arabian Gulf Catfish: A Unique Furan Fatty Acid (F6) and Cholesta-3,5-Diene (S5) Recruit Neutrophils and Fibroblasts to Promote Wound Healing

**DOI:** 10.3389/fphar.2020.00899

**Published:** 2020-06-18

**Authors:** Jassim M. Al-Hassan, Aleksander Hinek, Waleed M. Renno, Yanting Wang, Yuan Fang Liu, Rui Guan, Xiao-Yen Wen, Michael L. Litvack, Andras Lindenmaier, Mohammad Afzal, Bincy Paul, Sosamma Oommen, Divya Nair, Jijin Kumar, Meraj A. Khan, Nades Palaniyar, Cecil Pace-Asciak

**Affiliations:** ^1^Department of Biological Sciences, Faculty of Science, Kuwait University, Kuwait City, Kuwait; ^2^Program in Translational Medicine, Peter Gilgan Centre for Research and Learning (PGCRL), The Hospital for Sick Children, Toronto, ON, Canada; ^3^Department of Anatomy, Faculty of Medicine, Kuwait University, Kuwait City, Kuwait; ^4^Zebrafish Centre for Advanced Drug Discovery & Keenan Research Centre for Biomedical Science, Li Ka Shing Knowledge Institute, St. Michael’s Hospital, Unity Health Toronto, Toronto, ON, Canada; ^5^Departments of Lab Medicine and Pathobiology, and Institute of Medical Sciences, Faculty of Medicine, University of Toronto, Toronto, ON, Canada; ^6^Department of Zoology, CMS College, Kottayam, India; ^7^Department of Pharmacology, University of Toronto, Toronto, ON, Canada

**Keywords:** furan F-acid, cholesta-3,5-diene, Fraction-B, Gulf catfish lipids, wound healing, histology, fibroblasts, leukocyte

## Abstract

Preparations from Arabian Gulf catfish (*Arius bilineatus*, Val) epidermal gel secretion (PCEGS) effectively heal chronic wounds in diabetic patients. However, specific lipid components of PCEGS that are responsible for various aspects of wound healing are unknown. Here, we report for the first time that, i) a unique preparation containing only proteins and lipids (Fraction B, FB), derived from the PCEGS accelerated the healing of experimental dermal wounds in female rats (transdermal punch biopsy) *in vivo*. Histological analyses showed that topical treatment of these wounds with FB promoted the migration of fibroblasts, facilitated the production of extracellular matrix (collagen, fibronectin), induced capillary formation and recruitment of immune cells, and accelerated overall wound healing by day 4 (tested at 1, 2, 3, 4, and 10 days; n=15 for vehicle; n=15 for FB treatment), ii) the lipids responsible for different stages of wound healing were separated into a protein-free bioactive lipid fraction, Ft, which contained a few common long-chain fatty acids, a unique furan fatty acid (F6) and a cholesterol metabolite, cholesta-3,5-diene (S5). Ft (the partially purified lipid fraction of PCEGS), and F6 and S5 present in Ft, proved to be bioactive for wound healing in human dermal fibroblasts. Ft increased the production and extracellular deposition of collagen and fibronectin, *ex vivo*, iii) Ft and its subcomponents, pure F6 and S5, also promoted human dermal fibroblast migration into the scratch wound gaps, *ex vivo*, iv) Ft, F6, and S5 promoted the recruitment of neutrophils (Green fluorescence protein labeled) to the site of injury in the transected tailfins of transgenic zebrafish, *in vivo*, v) Ft, but not F6 or S5, promoted the regeneration of tissues at the wound site in the transgenic zebrafish tailfin, *in vivo*. Therefore, we conclude that lipid fraction Ft from PCEGS contains the components necessary to promote complete wound healing, and F6 and S5 are responsible for promoting fibroblast and neutrophil recruitment to the site of wounds.

## Introduction

Stressed or wounded scale-less Arabian Gulf catfish (*Arius bilineatus*, Val.) quickly release a gel-like substance (PCEGS) from their skin (Al-Hassan and Criddle, 1982; Al-Hassan et al., 1987). These fish that are often wounded by marine predators, developed the unique mechanism by which they are able to effectively heal their wounds (Al-Hassan et al., 1985). This curious observation initiated a series of studies directed by one of us [JA-H] (Al-Hassan et al., 1983; Al-Hassan et al., 1991) that established PCEGS preparations could accelerate wound healing (Al-Hassan et al., 1983; Al-Hassan et al., 1991), and were found effective in treating human chronic diabetic foot ulcers that were unresponsive to conventional therapy (Al-Hassan, 1990). Biochemical analysis has shown that the PCEGS preparations, used in the initial studies, contained several biologically active proteins, including growth factors (Al-Hassan et al., 1986; Al-Lahham et al., 1987; Thomson et al., 1989) and lipids (Al-Hassan et al., 1986; Plattner et al., 1988; Summers et al., 1991; Al-Hassan et al., 1998; Khan et al., 2018; Al-Hassan et al., 2019). Fraction B with its lipid contents, being the center of our research activities, and since the dermal preparations were rich in lipids, we set out to identify other active putative lipid ingredients in PGCES that contribute to various stages of wound healing.

We particularly focused on fractionation and purification of various lipids, and tested their putative biological activities using an experimental animal dermal wound healing model (*in vivo*), human dermal fibroblast proliferation and scratch wound healing assays (*ex vivo*). We also tested whether these preparations would facilitate migration and/or proliferation of human dermal fibroblasts in the scratch gaps of wounded monolayer cultures (*ex vivo* model) and neutrophil migration to the site of injury in a transgenic zebrafish transected tailfin wound healing model (*in vivo*). Data presented in this paper show that specific lipids present in Ft lipid fraction derived from PCEGS play major roles in the wound healing processes, and that two lipid components of Ft (F6 and S5) promote neutrophil recruitment at the site of the wounds. These findings assigned novel roles for F6 and S5 that could encourage their use in therapeutic formulations for the efficient healing of human wounds.

## Materials and Methods

### Biochemical and Lipids

Lipids present in the PCEGS preparations were extracted and separated by HPLC, and identified by GC-MS, as described previously (Khan et al., 2018; Al-Hassan et al., 2019). Pure F6 was purchased from Cayman Chemicals, Ann Arbor, MI. Pure S5 was purchased from Sigma-Aldrich (Burlington, MA). All the other biochemical reagents were purchased from Sigma, unless otherwise stated.

### Experimental Dermal Wound Healing in Rats

All animal procedures were performed in accordance with the National Institutes of Health (NIH) Guidelines for the Care and Use of Laboratory Animals and the Animal Facility at Health Science Center, Kuwait University. Female Sprague Dawley rats (n=30) approximately two months old were used, each weighing about 150 g. They were kept under controlled temperature (23 ± 2°C) and light (12-h light-dark cycle) with free access to food and water. The animals were randomly divided into two groups with 15 animals in each group. The experimental protocol started when the animals underwent the surgical procedure (day 0). The animals were intraperitoneally anesthetized with 50 mg/kg Ketamine^®^ and 10 mg/kg Xylazine^®^. The dorsal surface was shaved and cleaned with alcohol. The central upper back skin was manually pulled up at the midline and two subcutaneous surgical wounds were made using a 4 mm disposable biopsy punch (ACU PUNCH 4.0 MM (CONF. 20), DIFA PROCARE) on both right and left lateral sides of the midline (1-2 cm apart from the midline). Animals with wounds were divided into two groups. Group 1: control, treated with normal saline; Group 2: treated topically with Fraction-B (FB), 3 mg/kg every day up to day 10.

The animals were placed in individual cages and were monitored until full recovery. The drug treatment was applied topically from day 1 to day 10 according to the groups and their sacrifice date. In addition to the daily treatment after surgery, all animals were monitored and sacrificed according to the following protocol: 3 rats per group on day 1, 2, 3, 4, and 10. Wounds were photographed for gross morphological evaluation (Canon EOS 600D, ISO Speed—1600, F-stop 7.1) before the skin wound specimens were dissected out for histological examination. Skin samples from the two wounded areas were immediately fixed in 4% (v/v) paraformaldehyde (PFA) for overnight at 4°C and later washed with 0.1 M phosphate buffered saline (PBS; pH 7.4). The skin specimens were processed for routine paraffin embedding protocol (Külkamp-Guerreiro et al., 2013; Perini et al., 2015), and histological analyses.

### Scratch Wound Healing Assay Using Human Dermal Fibroblasts

The approval of our Institutional Ethics Review Board and patient informed consent were obtained for all described studies that used small fragments of the healthy skin collected during plastic surgery procedures. Guidelines for the protection of human subjects of the Department of Health and Human Services and of the Declaration of Helsinki Principles were followed in obtaining tissues for this investigation. Biological effects of the lipid preparation Ft and pure F6 and pure S5 were tested in secondary cultures of dermal fibroblasts (cell passages 3-5), derived from three healthy Caucasian females (26-, 30-, and 50 years old). All fibroblasts were originally isolated by trypsinization of skin biopsies with 0.25% (w/v) collagenase type I and 0.05% trypsin/EDTA and then maintained in alpha-minimum essential medium supplemented with 20 mM 4-(2-hydroxyethyl)-1-piperazineethane sulphonic acid (HEPES), 1% antibiotics and antimycotics, 1% L-glutamate, and 2% FBS as previously described (20-22). In all experiments, consecutive passages 3–5 were tested.

Cover slips were placed on 6-well tissue culture dishes. Freshly trypsinized fibroblasts (25 × 10^4^) were dispersed in culture medium and plated onto the coverslips and incubated at 37°C for 30 h in the presence of 5% (v/v) CO_2_, in the confluent monolayers; identical straight-line central scratches were created in multiple cultures, using a standard p1000 pipet tip. The cell debris was washed off, and 2 ml DMEM medium containing 1% (v/v) FBS was added. The tested compounds (Ft, pure F6 and pure S5) were added at indicated concentrations to all cultures and further incubated for the next 7, 24, or 48 h. The cultures were placed under the phase-contrast microscope (Olympus CKX41) and their initial and timed scratched regions were photographed by phase-contrast microscope attached with Lumenera's Infinity 1 camera. The cell migration rates into the scratched areas were analyzed; using Adobe Photoshop and Microsoft office excel programs.

The proliferation rates of scratch-filling cells were also analyzed in 48-h-old cultures. The cells were fixed with 100% ice-cold methanol at -20°C for 30 min and incubated for 1 h with polyclonal antibody to ki67 proliferative antigen and consecutively with the fluorescein-conjugated secondary antibody (GAR-FITC, Sigma Aldrich) for an additional hour. Cell nuclei were counterstained with propidium iodide. Pictures of scratch areas were taken using a Nikon Eclipse E1000 microscope equipped with a cooled charge-coupled device camera and NIS Element imaging analysis, then cell proliferation was analyzed with ImageJ software and Microsoft office excel.

### Digital Analysis of Fibroblast Migration by Automated Cell Tracking

We applied a digital software assisted measurement strategy to quell some of the limitations of the standard migration assay measurements. This was accomplished by using digital image analysis and automated cell tracking through image segmentation. Standard greyscale images were segmented into black (cell culture media—negative space) and white (cell population—positive space) pixels using gradient magnitude quantification in MATLAB R2017 (MathWorks, Natic, MA). Cell area was defined as white, whereas non-cellular area was defined as black. Edges of cells were identified prior to binarization and coded to be associated with cells and defined as white pixels. The binarized images were evaluated using an automated segmentation and quantifying program designed to measure black-to-white (B/W) pixel ratio. For each 1600 x 1200-pixel image area, column-wise B/W pixel ratio was calculated from the image 1600 pixel columns. The column ratios were measured for images taken immediately after the cellular monolayer was disrupted (e.g., 0 h), followed by measurement at 24 h and 48 h. All cellular monolayer samples were re-centered based on the center column of the disruption region (i.e. black-only column of pixels at the initial 0 h time-point). This centring technique allowed us to average images taken of the different replicates and track them at the different times and regions to a normalized center. Thus, the data measurements could be compared amongst a variety of images regardless of the position of the disruption within the individual image.

### Effect of Ft on the Production of Collagen-I and Fibronectin by Human Dermal Fibroblasts

All cell culture products were obtained from GIBCO Life Technologies (Burlington, Ont., Canada). Cultures of fibroblasts, which produce abundant ECM, were used. Three-day-old cultures grown in the presence of vehicle or Ft were fixed in 100% (v/v) ice-cold methanol and blocked with 1% (v/v) normal goat serum. Cultures were incubated for 1 h, either with 2 µg/ml of polyclonal antibodies to collagen type I (a generous gift of Larry W. Fischer from The National Institute of Health, Bethesda, MD), or with 1 µg/ml of monoclonal antibody to fibronectin (mAB1940, Chemicon Temecula, CA). All cultures were incubated for an additional hour with appropriate fluorescein-conjugated secondary antibodies (GAR-FITC or GAM-FITC, Sigma). Collagen and Fibronectin were immune-detected with specific antibodies as stated, and the nuclei were counterstained with propidium iodide (PI; red) or 4',6'-diamidine-2'-phenylindole dihydrochloride (DAPI; blue). Morphometric analysis of cultures immunostained with antibodies recognizing extracellular matrix components was also performed using a computerized video analysis system (Image-Pro Plus software 3.0, Media Cybernetics, Silver Spring, MD) as described previously (Hinek et al., 2000).

### Neutrophil Migration and Tailfin Regeneration in Transgenic Zebrafish

Zebrafish strain AB, Tg(mpx:GFP) were raised and maintained using standard laboratory procedures (Avdesh et al., 2012). Embryos were obtained *via* natural mating and cultured in embryo E2 medium (Nüsslein-Volhard and Dahm, 2002) and incubated at 28°C ± 0.5°C. All experiments in the study were conducted according to the ethical guidelines established by the St. Michael's Hospital Animal Care Committee and Research Ethics Board with approved animal protocol ACC660. Test compounds were dissolved in ethanol (2 µl) and diluted with 300 µl E2 medium. The zebrafish line in which neutrophils are labelled with green fluorescence, were used to investigate the neutrophil migration. Fish larvae with tailfin uncut were maintained in 24-well plastic dishes. Each well contained 10 larvae in 700 µl of E2 medium before addition of test compounds in the same medium. Compounds were added to each well right after tailfin transection. Following treatment, neutrophil migration and tailfin regeneration were assessed at certain time points. Non-injected controls were included in separate wells on every dish. Fish larvae at 4 days post fertilization (dpf) were anesthetized in E2 medium containing 0.1 mg/ml Tricaine before wounding. Tailfin transection was performed with a 30-gauge sterilized needle, using 70% (v/v) ethanol prior to use. A single cut was made traversing the entire dorsoventral length of the caudal fin, posterior to muscle and notochord in each fish. For neutrophil migration the larvae were incubated to 6 hpa (hours post-amputation) at 32°C. Before imaging with fluorescence stereomicroscope (Leica M205 FA), the larvae were anesthetized with 0.1mg/ml Tricaine. Neutrophil numbers at the site of tail wound were counted using Fiji software.

### Statistical Analysis

Data are expressed as mean ± standard error. Comparison between multiple groups was performed by ANOVA followed by Tukey's post-hoc test. Pairwise comparisons were performed by t-test. A p-value of ≤ 0.05 was considered to represent statistically significant differences between samples.

## Results

### Fraction B (FB) of PCEGS That Contains Proteins and Lipids, Promotes Rat Skin Wound Healing, *In Vivo*

Previous studies showed that FB (containing lipids and proteins) prepared from PCEGS effectively healed human diabetic foot ulcers (Al-Hassan et al., 1987). However, the wound healing response exerted by FB was not established in detail. Therefore, in this study, we first used an *in vivo* model of experimental skin wounding in healthy female rats to study the effect of FB on the healing of those wounds. We needed to know whether FB could accelerate wound healing in animals. These experiments were necessary to further explore/identify factors present in FB, using *in vitro* and *in vivo* models. We generated round wounds, penetrating the whole skin thickness of the anesthetized rats, using a sharp punching pipe tool. These punched wounds were then treated once a day, either by a topical administration of PBS (control; n=15) or by Fraction-B (FB; 3 mg/kg; n=15) derived from PCEGS.

**Figure 1:**
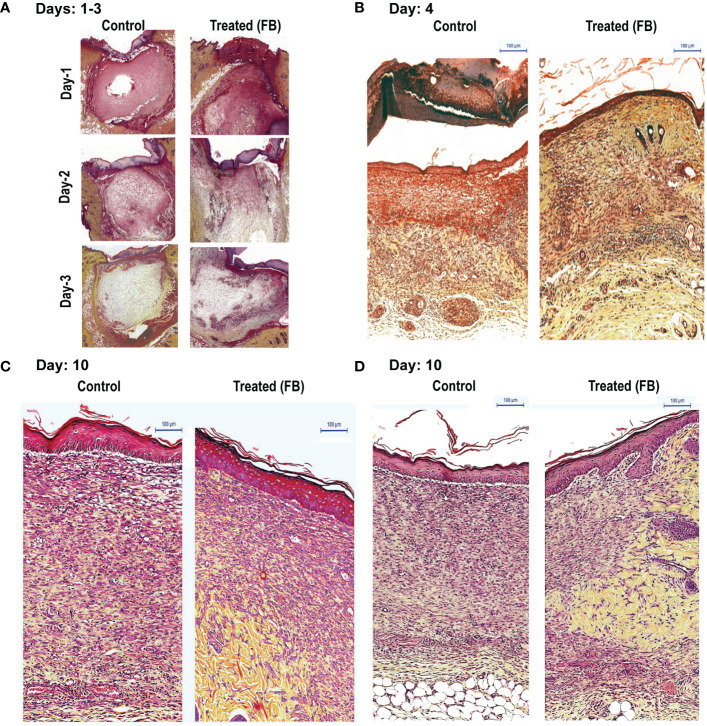
Dermal tissue histology shows that topical application of FB promotes wound healing in rats, *in vivo*. Images show the histopathology of sections (Movat’s stain) of skin punch biopsy on days 1, 2, 3 **(A)**, 4 **(B)**, and 10 **(C, D)**, after daily wound treatment with the PCEGS derived Fraction-B (proteins + lipids). Note in the FB-treated animals enhanced collagen deposition (yellow), new connective tissue containing more extracellular matrix with yellow collagen, more leukocytes (round cells) and fibroblasts (elongated cells with space in between containing yellow collagen) and more mature capillaries (n = 3, in each condition, and total 30 rats).

To determine the consecutive stages of the healing process, after determining the gross morphology of the wounds visually, we prepared thin histological sections, from the wounded skin samples collected (three PBS-treated and from 3 FB–treated rats; at 1, 2, 3, 4 and 10 days after wounding). Wound morphology was examined after staining the specimen with the Movat's method. Histologic analysis of tissue sections demonstrated wounds of rats from experimental groups, which were initially (days 1 to 3) covered by the coagulation crusts (**Figure 1A**). Those crusts were then, either dissolved or partially detached from the wound surfaces after 4 days (**Figure 1B**). Also, all of the 4-day-old wounds (treated and untreated) were already covered by a new epithelium (**Figure 1B**). The wounded areas (located under the epithelium) were filled by the proportionally increased volume of new granulation tissue that steadily filled up the entire space between healthy skin edges, in both groups. Remarkably at all observed stages, experimental wounds treated with FB, were covered with the better developed epithelium that contained more cellular layers than their PBS-treated control counterparts. While, even at a very early stage (day 1) the granulation tissue contained a large number of mononuclear leukocytes infiltrating the amorphous tissue remnants. This tissue has been steadily dissolved during the 3 days, creating the empty holes around the single leukocytes, in both control and the FB treated groups. Later, (between day 4 and 10), in contrast to the PBS treated wounds that contained the large volume of new granulation tissue, the FB-treated wounds demonstrated a decreased volume of the granulation tissue-filled areas. More, precisely, the granulation tissue developing in untreated wounds consisted of numerous leukocytes, very densely packed fibroblasts, surrounded only by scarce collagen fibers (stained yellow) and proteoglycans (stained green). They also contained few newly formed capillaries. Therefore, histology of this tissue reflected the active inflammatory and proliferating stage of the wound healing. In contrast, the granulation tissues filling of the FB-treated wounds (at a comparable time stage) reflected a more mature stage of wound healing (**Figure 1C**). They had fewer leukocytes and more elongating fibroblasts and myo-fibroblasts, surrounded by a higher volume of the newly deposited collagen and fibronectin, especially located in the deeper part of the wounds. The FB treatment also resulted in penetration of more frequent and larger capillaries than their PBS-treated counterparts. It is also important to mention that at the later stages of their healing (day 10) in the FB-treated wounds, the initial granulation tissue has been replaced by the new fibrillar connective tissue containing a new collagen and fibronectin, augmenting from the healthy wound edges. The FB-treated wounds further demonstrated that their newly generated tissue also contained multiple macrophages, engaged in removal of the initial granulation tissue debris for resolving the wounds (**Figure 1D**, **Supplementary Figure 1S**).

### Lipid Fraction From PCEGS Promotes Collagen and Fibronectin Deposition in the Cultured Fibroblasts, Ex Vivo

**Figure 2:**
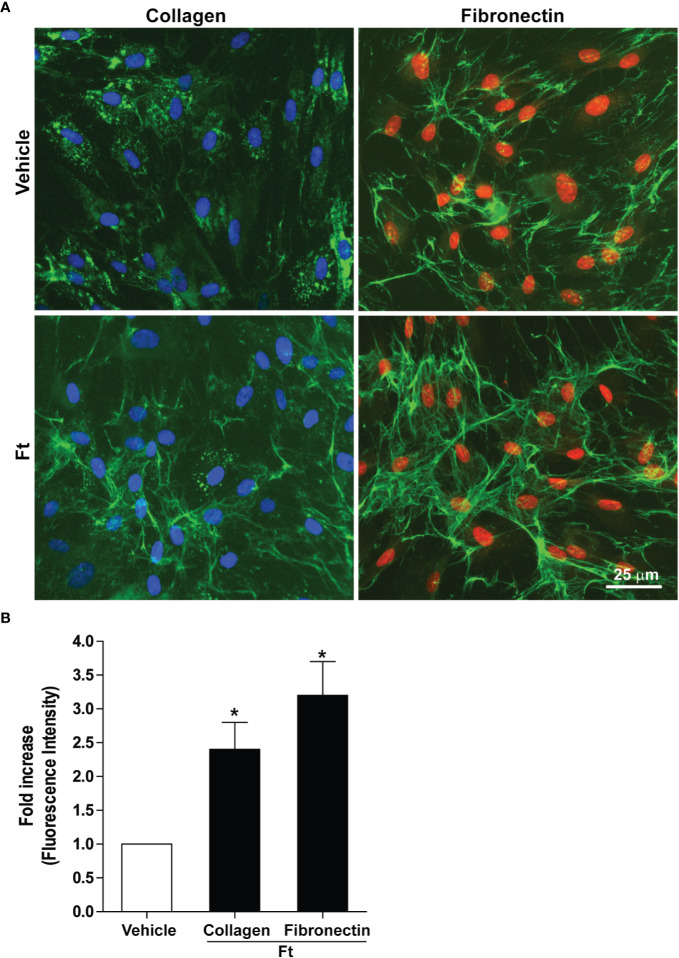
Ft promotes the production of extracellular matrix components by human fibroblasts, *ex vivo*. **(A)** Collagen and fibronectin (green fluorescence) from vehicle- and Ft-treated fibroblast cultures. **(B)** Quantitative data show the effect of Ft on increasing level of fibroblast collagen and fibronectin from these experiments (n=3, 5–6 images were analyzed in each experiment, p < 0.05; *, significantly different compared to the control).

Two of the key events necessary for wound healing are the deposition of extracellular collagen and fibronectin (Ruszczak, 2003; Zollinger and Smith, 2017; do Amaral et al., 2019; Wang et al., 2019). Hence, to determine the effect of the bioactive lipid fraction, Ft, on collagen and fibronectin deposition, we used human dermal fibroblasts isolated from three donors. We treated these fibroblasts with vehicle as controls or Ft (12.5 µg/ml) for 48 h. Immunostaining followed by fluorescence microscopy showed that, compared to the vehicle controls, Ft promoted the deposition of both components (**Figure 2A**). Quantitative analyses showed that Ft promoted the production of >2-fold more collagen and >3-fold more fibronectin (**Figure 2B**). Therefore, Ft has the ability to promote the production and deposition of extracellular matrix components from fibroblasts. This ability of Ft could effectively lead to acceleration of the wound healing processes.

Another key requirement for the efficient wound healing is the proliferation of fibroblasts (Gunin et al., 2018). To determine whether Ft also promotes fibroblast proliferation, we treated the dermal fibroblast obtained from three donors with vehicle control or 12.5 µg/ml Ft for 48 h. The cells were fixed and stained for Ki67, a marker for cell proliferation. This marker is absent in the resting cells, but present in the nuclei of proliferating cells (Sobecki et al., 2016). Our image analyses demonstrated that the 2 days-old Ft-treated cell cultures of human dermal fibroblasts contained similar levels of the immuno-detectable (green) proliferating antigen as their untreated counterparts (**Supplementary Figure 2S**). Therefore, we conclude that this Ft fraction is more capable of promoting extracellular matrix production than fibroblast proliferation.

### Lipid Fraction Promotes Fibroblast Migration Into the Wound Gap, Ex Vivo

**Figure 3:**
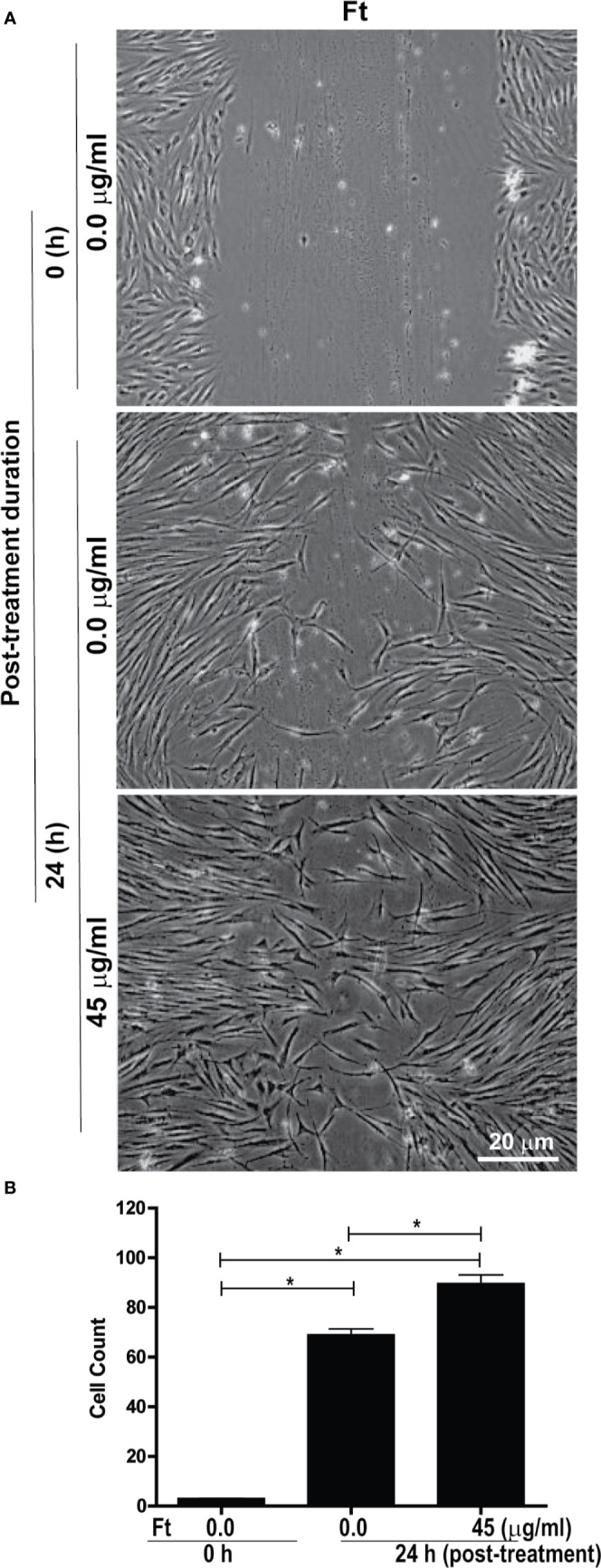
Ft promotes fibroblast migration into the scratch wound, *ex vivo*. Assessment of migration of fibroblasts into a midline gap evoked by incubation with Ft. **(A)** photographs of the midline gaps in the presence of Ft or its vehicle control at gap initiation (0 h) and 24 h following incubation. Photographs show the transparent cells on a dark background. **(B)** Quantitative analyses show the cell migration into the scratch wound (n = 3, p < 0.05; *, significantly different, as indicated by horizontal bars).

Another major step in wound healing is the fibroblast migration into the wounded area. Therefore, we tested whether Ft could stimulate fibroblast migration using a classical scratch wound healing assay. Gap measurements at 24-h time point showed that Ft (45 µg/ml) enhanced the migration of fibroblasts into the scratch gap (**Figure 3**). Hence, we conclude that Ft contains fibroblast migration promoting activity.

### F6 and S5, Two of the Lipid Components of Ft, Promote Fibroblast Migration Into Wound Gap, Ex Vivo

**Figure 4:**
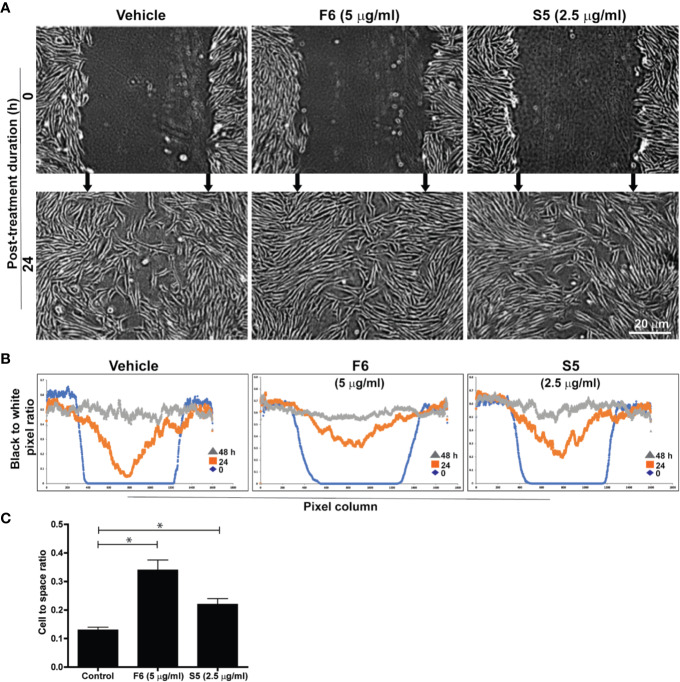
F6 and S5 promote fibroblast migration into the scratch wound, *ex vivo*. Assessment of migration of fibroblasts into a midline gap evoked by incubation with furan fatty acid F6 and the steroid S5 at indicated doses. **(A)** photographs of the midline gaps in the presence of the two compounds (S5 or F6 and their vehicle control) at gap initiation (0 h) and 24 h following incubation. Photographs show the transparent cells on a dark background with manual integration. **(B)** the number of cells infiltrated into the gap after 0, 24, and 48 h from binarized images using an automated segmentation and quantification program designed to measure black-to-white (B/W) pixel ratio (based on similar images shown on panel A). Blue lines represent the B/W pixel ratios for the 1600 columns of the images taken immediately following monolayer disruption (0 h). The red and grey lines describe the B/W pixel ratios for the 1600 columns of the images taken 24 h and 48 h following monolayer disruption, respectively. **(C)** Quantitative analyses of the data at 24 h in terms of cell to space ratio, show the fibroblast migration into the scratch wound (n = 3-4, p < 0.05; *, significantly different compared to the control).

We have recently reported that major components of Ft were long-chain fatty acids. Ft also contains a minor bioactive component, furan fatty acid (F6) (Khan et al., 2018; Al-Hassan et al., 2019) and a cholesterol metabolite, cholesta-3,5-diene (S5) as shown by GC-MS (**Supplementary Figure 3S**). Since our experiments suggested that Ft enhanced the migration of fibroblasts into the scratch gap (**Figure 3**), we conducted a similar assay to determine fibroblast involvement in wound healing with pure F6 and S5. The wound gap closure assays indicated that both F6 (5 µg/ml) and S5 (2.5 µg/ml) promoted wound closure by fibroblasts recruitment (**Figure 4A**). Since the simple measurements of distance of the gaps miss the growth differences that can have biological or mechanical value, we used digital image analyses with automated cell tracking through image segmentation. Such a quantitative analysis clearly showed that both F6 and S5 increased the number of fibroblasts migrating into the wound gap (**Figures 4B, C**). Therefore, we concluded that these two lipids F6 and S5, being key bioactive components of Ft, are responsible for promoting fibroblast migration into the injured gap during wound healing, in monoculture experiments, *ex vivo*.

### Lipid Fraction Ft Promotes Neutrophil Recruitment to the Sites of Wounds in Zebrafish, In Vivo

**Figure 5:**
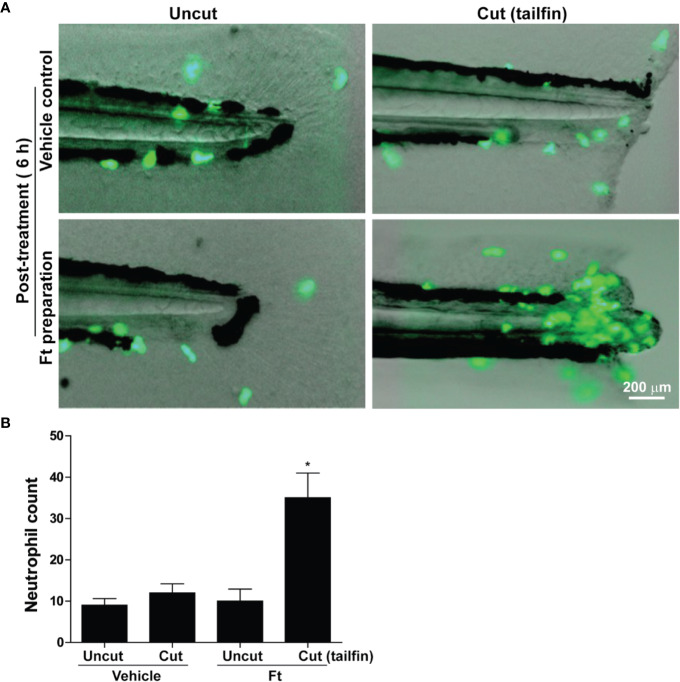
Ft promotes neutrophil recruitment to the tailfin injury sites of transgenic zebrafishes. **(A**) images showing the migration of green fluorescent protein labeled neutrophils in transgenic zebrafish larvae after tail fin transection with Ft (50 µg/ml) or vehicle treatment for 6 h. No specific neutrophil recruitment is detectable at the uncut tailfin fish tips. **(B)** Quantitative differences in neutrophil accumulation at the tip of the tailfin (n=10, p < 0.05; *, significantly different compared to the uncut control).

Leukocytes are another class of cells important for wound healing, *in vivo* (Bouchery and Harris, 2019; Kreimendahl et al., 2019). The initial stage of repair of wounded skin consistently involves the rapid recruitment of leukocytes, particularly neutrophils to the site of injury (desJardins-Park et al., 2018). An ideal model to study neutrophil recruitment to the sites of wounds is the transgenic zebrafish that carry green fluorescent protein (GFP) labeled neutrophils (Tg(mpx:GFP) (Renshaw et al., 2006). We wounded these transgenic zebrafishes, at the larval stage 4 (dpf), by the transection of their tailfins, and treated them with different concentrations of Ft (0-50 µg/ml; in the wells). The larvae were anesthetized and imaged using fluorescence microscopy. Remarkably, 6 hours after the Ft treatment, neutrophils had rapidly accumulated at the tailfin wound sites (**Figures 5A, B**). By contrast, the transected fins of the vehicle-treated counterparts attracted only a few infiltrating neutrophils at the same time point. Importantly, neutrophils did not accumulate in both Ft-treated and vehicle-treated uninjured control fish larvae, indicating that lipid components present in Ft promote neutrophil recruitment only to the site of wounds.

### F6 and S5, Two of the Lipid Components of Ft, Promote Neutrophil Recruitment to the Wound Site in Zebrafish Tailfins

**Figure 6:**
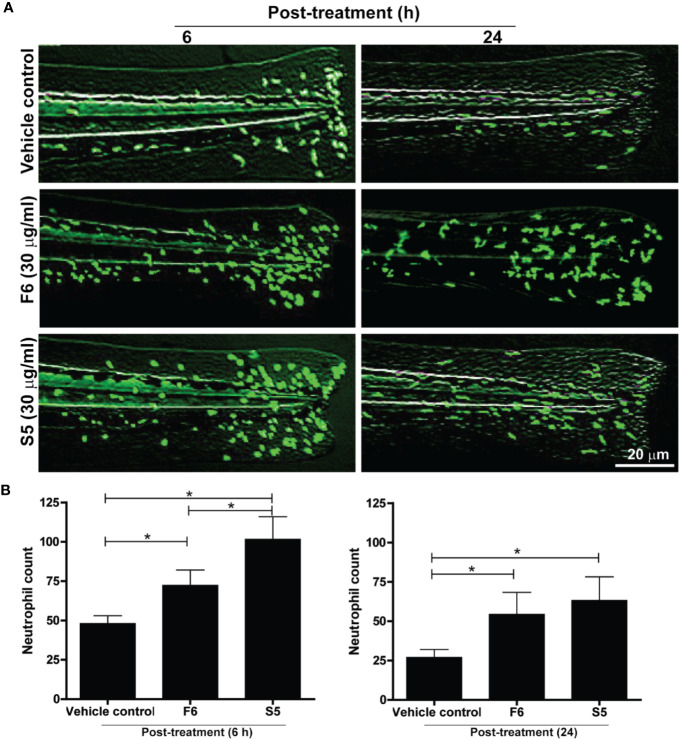
Two Ft components, F6 and S5, promote neutrophil recruitment to the tailfin injury sites of transgenic zebrafishes. **(A)** Fluorescence microscopy images show that F6 and S5 at 30 µg/ml cause enhanced migration of green fluorescent neutrophils to the dissected caudal fins in zebrafish larvae by 6 h and 24 h. **(B)** Quantitative analyses of neutrophil accumulation at wound sites (n=10, p < 0.05; *, significantly different, as indicated by horizontal bars).

Lipid components of Ft responsible for neutrophil recruitment to the site of wounds were unknown. Hence, we tested the effect of the two unique lipid components (S5 and F6) as potential candidates for exerting neutrophil recruitment, using transgenic zebrafishes that carry fluorescent labeled neutrophils (Tg(mpx:GFP). Tailfin dissected larvae were treated with various concentrations of F6 (0-30 µg/ml) and S5 (0-30 µg/ml), in the wells of microplates. Fluorescence stereo-microscopy of anesthetized larvae showed that both F6 and S5 had impressive ability to recruit neutrophils to the dissected tailfins, by 6 h (**Figure 6A**). Quantitative analyses revealed that these compounds (30 μg/ml) promoted the accumulation of neutrophils at the site of the wound, by >2 fold compared to the vehicle control-treated larvae (**Figure 6B**). At similar concentrations, S5 showed a more potent effect than F6 at 5 h; and the effect was similar for F6 and S5 at 24 h (**Figure 6B**). There was no neutrophil migration in response to these compounds in uninjured tailfins (data not shown). Therefore, we concluded that these two lipid components facilitated neutrophilic immune response during the initial stages of wound healing, *in vivo*.

### Ft, but Not F6 and S5, Was Sufficient to Promote Regeneration of Wounded Zebrafish Tailfins

**Figure 7:**
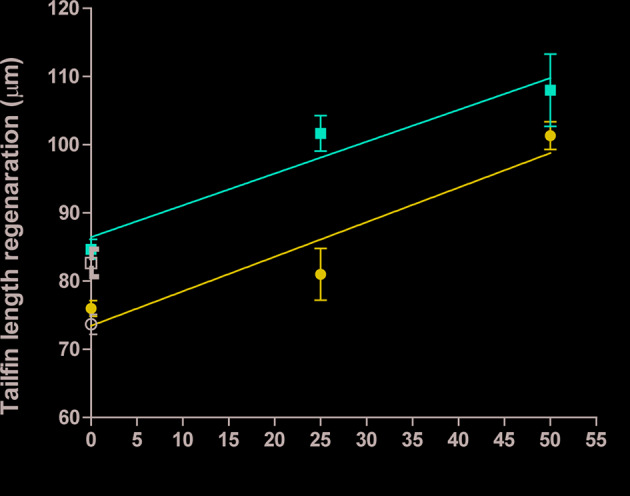
Ft promotes regeneration of injured tailfin in transgenic zebrafishes. Quantitative analyses of the regeneration of tailfin in freshly dissected zebrafish tailfins after days 2 and 6. Indicated p-values (< 0.05) show that the slopes are more than 0 (n = 10).

To determine whether the lipid-mediated neutrophil recruitment facilitate tissue regeneration, *in vivo*, we next added Ft, pure F6, or pure S5 to the tailfin dissected Tg(mpx:GFP) zebrafish larvae, and measured the extent of tissue regeneration at days 2 and 6 after treatment (n=10). Ft dose-dependently increased tailfin regeneration (**Figure 7**), indicating that Ft contained lipid components other than F6 and S5 that played important roles in the tissue regeneration. However, as F6 (0-10 µg/ml) and S5 (0-5 µg/ml), a concentration that is expected to be 10x higher than found in Ft, did not promote significant tissue regeneration (**Supplementary Figure 4S**), their presence in Ft plays a unique regulatory role in the neutrophil recruitment stages of wound healing but not in the tissue regeneration stage, *in vivo*.

## Discussion

Previous studies conducted by one of us (JMAH) demonstrated that topical application of preparations from catfish epidermal gel secretions (PCEGS) from *Arius bilineatus*, Val, containing proteins and lipids, secreted by stressed scale-less catfish accelerated healing of human diabetic foot ulcers that were unresponsive to conventional treatments (Al-Hassan et al., 1983; Al-Hassan, 1990). Crude PCEGS showed the presence of several biologically active proteins (Al-Hassan and Criddle, 1982; Al-Hassan et al., 1986; Al-Hassan et al., 1987) and growth factors (Al-Hassan et al., 1991) that have been implicated in wound healing in animal and human tissues. In this study, we investigated whether lipid molecules present in the bioactive PCEGS could contribute in the overall wound healing processes. These studies show that F6 and S5 contribute to a number of biological activities attributable to a lipid sub-component of PCEGS.

We first examined the effect of FB (containing proteins and lipids) on dermal wound healing on female rats, *in vivo*. Histology of the granulation tissue filling the PBS-treated wounds at early stages (Days 1-4) reflected the active inflammatory and proliferating stage of the wound healing. By contrast, the granulation tissues filling the FB-treated wounds reflected a more mature stage of wound healing. They had fewer leukocytes and more elongating fibroblasts and myo-fibroblasts, surrounded by a higher volume of the newly deposited collagen and fibronectin, especially located in the deeper part of the wounds (**Figures 1A, B**). We concluded that components present in FB not only accelerated the healing process, but changed its character toward the early fibrotic response, that was executed by the already existed, migrating fibroblasts, not by the next generation of proliferating cells. While the involvement of numerous platelet derived growth factors and those released from the wound crust cannot be excluded from the observed healing process, we speculate that chemotactic abilities of the lipid components of FB play an equally important role in attraction of the debris-removing macrophages and the ECM producing fibroblast into the central parts of the wound, ultimately changing its character from just inflammatory response, into the pro-fibrotic process, likely stimulated by the consequently released endogenous growth factors such as PDGFs, TGFs and others, and the subsequent release of fibrous structures responsible for the mechanical holding and support of the wounded skin (Tymen et al., 2013; Barrientos et al., 2014; Bae et al., 2014; Pastar et al., 2014; Lam et al., 2015; Larouche et al., 2018; Rodrigues et al., 2019).

Collectively, our results indicate that treatment with FB enhanced several stages of the wound healing. Even though the initial wounding induced bleeding and accumulation of the temporary coagulation crust formation, in both experimental groups, FB treatment clearly facilitated a more efficient final wound healing (Day 10) (**Figures 1 C, D**). This was likely due to the initial FB-dependent activation of the resident fibroblast and neutrophils, as well as the concomitant stimulation of their chemotactic migration from the wound edges into the central parts of the initial wounds. This contributed to the clearing of the wounded tissue debris and accelerated the formation of the new structural connective tissue.

Wound healing is a complex process that involves extracellular matrix components, fibroblasts and immune cells such as neutrophils and macrophages (Yoon et al., 2018; Bouchery and Harris, 2019; Kreimendahl et al., 2019; Paganelli et al., 2020). The first set of our studies focused on establishing the wound healing effects of FB (lipids + proteins), *in vivo*, using an experimental rat model. Parallel to the previous studies in man (Suhaeri et al., 2018), histological analyses demonstrate that experimental dermal wounds in healthy female rats are initially covered by crusts of blood clots but heal well by day 10 (**Figure 1**). By contrast, treating wounds with FB promotes acceleration of wound healing with the deposition of increased amounts of collagen and fibronectin, and increased numbers of fibroblasts, macrophages and large capillaries. These are typical features of late stages of wound healing (Raekallio and Viljanto, 1983; Pettet et al., 1996; Templin et al., 2009; Suhaeri et al., 2018; Zheng et al., 2018; Opneja et al., 2019). These *in vivo* wound healing data suggested the presence of healing stimulatory component(s) in the FB preparations, as we have described previously in other wound healing models (Al-Hassan and Criddle, 1982; Al-Hassan et al., 1983; Al-Hassan JM et al., 1985; Al-Hassan et al., 1986; Al-Hassan et al., 1987; Al-Hassan et al., 1991). *Ex vivo* human fibroblast experiments with a bioactive lipid fraction Ft showed that it had the capability to increase the extracellular matrix, collagen and fibronectin, deposition from fibroblasts, but not fibroblast proliferation (**Figure 2**; **Supplemental Figure 2S**). Production of these two matrix components by fibroblasts is a hallmark of efficient wound healing (Mitts et al., 2005; Mitts et al., 2010; Sen et al., 2011). These results indicate that Ft contains multiple bioactive lipid components pertinent to the different stages in the wound healing. This phenomenon (tempering proliferation of the matrix-producing fibroblasts) occurring at the final stage of the healing process, might ultimately contribute to preventing hypertrophic scars formation (cosmetic effect), that often deteriorate the final appearance of wounded human skin. Such cosmetic effect was observed in the treated human wounds and ulcers (Al-Hassan JM et al., 1985, 5.

The lipid fraction Ft contains several common long-chain fatty acids and a furan fatty acid, F6 (Khan et al., 2018; Al-Hassan et al., 2019). Another component of Ft was identified as S5, a derivative of cholesterol (**Supplementary Figure 3S**). In pursuit of identifying bioactive lipid components in Ft that are involved in wound healing (**Figure 3**), we focused on the two unique lipid species, F6 and S5. Interestingly, both of these rare lipid molecules promote human dermal fibroblast migration into the scratch wound gaps, in monocultures *ex vivo* (**Figures 4** and **5**). This novel bioactivity was identified for F6 and S5. Our recent studies showed that F6 promotes the death of cancer cells by regulating cellular signalling and inducing apoptosis (Al-Hassan et al., 2019). Since F6 promotes fibroblast migration and death in cancer cells, this lipid species carries cell-specific activities in different types of cells immune cells such as neutrophils are essential for recognizing injured cells while macrophages are essential for removing dead cells from the wounds (Yoon et al., 2018; Bouchery and Harris, 2019; Opneja et al., 2019). FB treatment promoted macrophage recruitment to the rat dermal wound sites, *in vivo* (wound resolving late stages; **Figure 1**). Neutrophils are the first responders of tissue injury (Yoon et al., 2018; Bouchery and Harris, 2019). Several lipid molecules could act as neutrophil chemoattractants (e.g., LTB-4, hepoxilins; (Dho et al., 1990; McCormick, 2007; Szabady and McCormick, 2013; Debeuf and Lambrecht, 2018); however, these lipids are not present in Ft (Khan et al., 2018). Hence, we examined the importance of lipid components of PCEGS (Ft, F6, S5) in the context of neutrophil recruitment to the wound site. Transgenic zebrafish models with fluorescently labeled neutrophils are amenable for identifying molecules regulating neutrophil recruitment to the wound sites, in drug testing studies (Renshaw et al., 2006). Our findings showed for the first time, that Ft, F6, and S5 are effective recruiters of neutrophils to the wound sites (**Figures 5** and **6**). Our recent study showed that pure F6 effectively induces neutrophil extracellular trap (NET) formation in human neutrophils (Khan et al., 2018). NETs promote recruitment of neutrophils (Pettet et al., 1996; Templin et al., 2009; Zheng et al., 2018). Although detection of NET formation in zebrafish has not been clearly established, increased local NET formation in the presence of F6 could lead to increased neutrophil recruitment to wound sites. F6 and S5 may be important regulators of tissue inflammation during wound healing.

**Figure 8:**
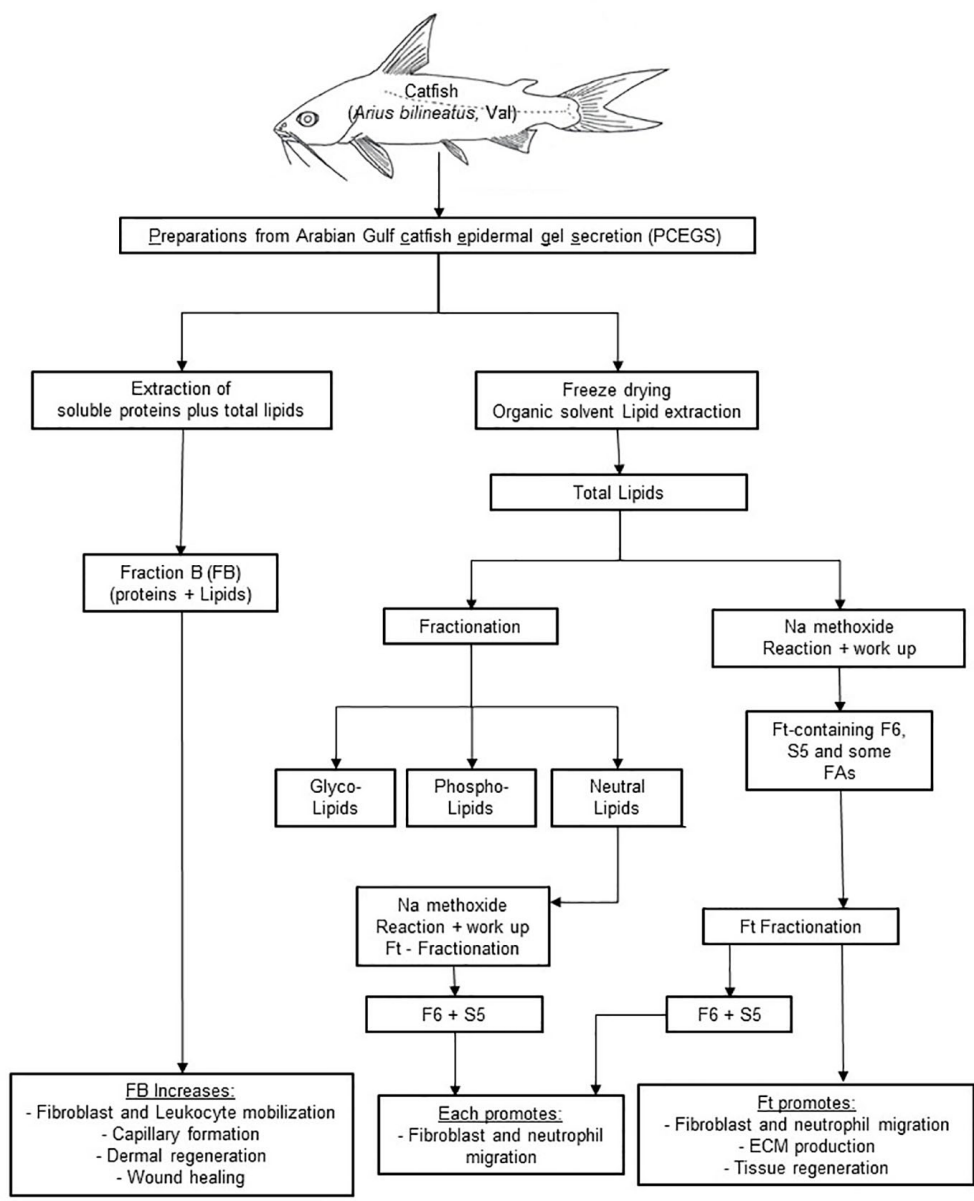
A scheme showing cascades of steps leading to application of FB, Ft, F6, and S5 from epidermal secretions of the catfish in wound healing. PCEGS preparations were fractionated into FB that contained proteins and lipids. Total lipid preparations derived from PCEGS were further fractionated into Ft. This study used a biologically active lipid fraction, Ft, containing its subtractions F6 (or furan fatty acid) and S5 (or cholesta-3,5-diene). Commercially available pure F6 and S5 were used to confirm their biological properties, as enlisted at the last row of the summary diagram.

In the experimental zebrafish fin transection model, Ft, but not F6 or S5, promotes effective tissue regeneration in wounded tissue, indicating the presence of other lipid factors in Ft that also contribute to tissue regeneration (**Figure 7**; **Supplementary Figure 4S**). Therefore, the data presented here indicate that these lipid preparations (Ft lipid fraction and pure F6 and S5 sub-fractions), may be excellent candidates for further testing either alone or in combination with other factors that have been previously implicated in the acceleration of wound healing. F6 and S5 are present in minor amounts in the lipid mixture of Ft, and even smaller amounts in the fraction FB. Hence, supplementing F6 and S5 to bioactive preparations (FB and Ft) may enhance the actions of components from PCEGS for specific applications [i.e. pro-NETosis (Khan et al., 2018), anticancer (Khan et al., 2018; Al-Hassan et al., 2019), and wound repair (Al-Hassan et al., 1985) (**Figures 1****–****8**)].

In summary, our results show that i) a fraction of PCEGS containing lipids and proteins (Fraction-B, FB) accelerated healing of experimental dermal wounds in rats (**Figure 1**; **Supplementary Figure 1S**), ii) a lipid fraction from PCEGS (i.e. Ft) promoted extracellular matrix deposition (collagen, fibronectin; **Figure 2**), but not fibroblast proliferation (**Supplementary Figure 2S**), in human fibroblast monocultures, *ex vivo*, iii) Ft and two of its lipid components (F6 and S5; **Supplementary Figure 3S**) promoted fibroblast migration into the scratch wound gaps, in fibroblast monocultures, *ex vivo* (**Figures 3** and **4**), iv) Ft and its subcomponents F6 and S5 promoted neutrophil recruitment to the tailfin wounds of zebrafish (**Figures 5** and **6**) and v) Ft, but not F6 or S5, promoted tissue regeneration of wounded tailfins of zebrafish, *in vivo* (**Figure 7**; **Supplementary Figure 4S**). Collectively, the current studies identified that specific lipid fraction Ft of PCEGS, particularly its components F6 and S5, are important lipid bioactive components responsible for specific stages of wound healing properties of PCEGS (**Figure 8**).

The three potential candidate lipid preparations (Ft and its components F6 and S5) of the epidermal secretions from *Arius bilineatus*, Val possess properties that accelerate the healing of cutaneous wounds. There are other compounds in the total preparation, which may act in concert with the preparation/factors described herein and may act on other stages involved in the overall wound healing process. These lipids (Ft, F6, and S5) may play essential roles in wound healing processes together with the other components identified in PCEGS such as the hemagglutinating factor (Al-Lahham et al., 1987; Al-Hassan et al., 1991), the hemolytic factor, the platelet aggregating factors with their platelet derived growth factors (Al-Hassan, 1990; Lam et al., 2015; Rodrigues et al., 2019; Opneja et al., 2019), and the vasoactive components (Suhaeri et al., 2018; Paganelli et al., 2020). It can be envisaged that the total aggregate effects of all these active components are unique and may act in synergism to accelerate wound and diabetic ulcer healing processes (Al-Hassan, 1990; Zheng et al., 2018). The current study paves the way for making potent therapeutic formulations with minimal number of stable active ingredients for wound healing (Gomes et al., 2017; Tan et al., 2019)or wound dressing applications (Zhao et al., 2017; Qu et al., 2018; Liang et al., 2019; Liang et al., 2019).

## Data Availability Statement

All datasets generated for this study are included in the article/**Supplementary Material**.

## Ethics Statement

The approval of our Institutional Ethical (SickKids) Review Board and patient informed consent were obtained for all described studies that used small fragments of the healthy skin collected during plastic surgery procedures. Zebrafish experiments in the study were conducted according to the ethical guidelines established by the St. Michael's Hospital Animal Care Committee and Research Ethics Board with approved animal protocol ACC660.

## Author Contributions

CP-A and JA-H conceptualized the project. CP-A supervised the Toronto portion of the project together with AH. CP-A, AH, and NP interpreted the data and wrote the manuscript. YW was involved in the cell culture experiments and data analysis. WR and JA-H were involved in the rat wound healing experiments. JA-H was the PI of the KFAS project grant and discovered the presence of F-acid family and the cholesterol metabolite family in the epidermal secretion with his associates (MA, SO, BP, DN) and prepared the catfish skin secretion to obtain FB preparation (proteins and lipids) and Ft fraction used in this study. JA-H also contributed to manuscript editing. The zebrafish studies were carried out by RG, YL, and X-YW. ML and AL were involved in computer analysis of fibroblast migration *in vitro*. MK participated in data analyses, final figure generation, manuscript editing, finalizing, and revising. All authors contributed to the article and approved the submitted version.

## Funding

This work was supported by Kuwait Foundation for Advancement of Sciences (to CP-A and to JA-H) KFAS grant #2013-120701 A-C, and by Research Sector, Kuwait University, grant No. SLO4/09 to JA-H, for which we are grateful. NP and MK received funding support from Natural Sciences and Engineering Research of Canada (NSERC; RGPIN436250-13) and Canadian Institutes of Health Research (CIHR; MOP-111012) for identifying anti-inflammatory molecules.

## Conflict of Interest

CP-A holds patents on anti-cancer/anti-thrombotic/anti-inflammatory small molecules unrelated to this work. JA-H holds patents on the use of the Arabian Gulf catfish gel for treating various diseases. AH has various patents unrelated to this work.

The remaining authors declare that the research was conducted in the absence of any commercial or financial relationships that could be construed as a potential conflict of interest.
