# Supplementary material for: Potential Mechanism of Dermal Wound Treatment With Preparations From the Skin Gel of Arabian Gulf Catfish: A Unique Furan Fatty Acid (F6) and Cholesta-3,5-Diene (S5) Recruit Neutrophils and Fibroblasts to Promote Wound Healing
Source: Front Pharmacol. 2020 Jun 18;11:899. doi: 10.3389/fphar.2020.00899 (PMC7314935; doi:10.3389/fphar.2020.00899)
Supplement: Supplementary file 1 [file DataSheet_1.pdf]

Supplementary Figures

Potential mechanism of dermal wound treatment with preparations from the skin gel of Arabian Gulf catfish: A unique furan fatty acid (F6) and cholesta-3,5-diene (S5) recruit neutrophils and fibroblasts to promote wound healing

Jassim M. Al-Hassan[1], Aleksander Hinek[2], Waleed M. Renno[3], Yanting Wang[2], Yuan Fang Liu[2], Rui Guan[5], Xiao-Yen Wen[5,6], Michael L. Litvack[2], Andras Lindenmaier[2], Mohammad Afzal[1], Bincy Paul[1], Sosamma Oommen[4], Divya Nair[1], Jijin Kumar[3], Meraj A Khan[2], Nades Palaniyar[2,6] and Cecil Pace-Asciak[2,7,*]

1. Department of Biological Sciences, Faculty of Science, Kuwait University, P.O. Box 5969, Safat 13060, Kuwait; jassim5577@hotmail.com (JMA-H); mafzal@ku.edu.kw (MA); bincymaniyalil@gmail.com (BMP); divyajs2002@yahoo.co.in
2. Program in Translational Medicine, Peter Gilgan Centre for Research and Learning (PGCRL), The Hospital for Sick Children, Toronto, ON M5G 0A4, Canada; alek.hinek@sickkids.ca (AH); michael.litvack@sickkids.ca ; andras.lindenmaier@sickkids.ca (AL); yuanfang.liu@sickkids.ca (YFL); meraj.khan@sickkids.ca (MAK); nades.palaniyar@sickkids.ca ; cecil.pace-asciak@sickkids.ca (CP-A)
3. Dept. of Anatomy, Faculty of Medicine, Kuwait University, Kuwait, wrenno@hsc.edu.kw (WR); jijinbioinfo@gmail.com
4. Department of Zoology, CMS College, Kottayam 686001, India; sosamma@cmscollege.ac.in (SO)
5. Zebrafish Centre for Advanced Drug Discovery & Keenan Research Centre for Biomedical Science, Li Ka Shing Knowledge Institute, St. Michael's Hospital, Unity Health Toronto, Toronto, ON M5B 1W8, Canada; ruiguan2012@gmail.com (RG); x.wen@utoronto.ca (XYW)
6. Departments of Lab Medicine and Pathobiology, and Institute of Medical Sciences, Faculty of Medicine, University of Toronto, Toronto, ON M5G 0A4, Canada
7. Department of Pharmacology, University of Toronto, Toronto, ON M5S 1A8, Canada

**corresponding author*

Running title: A furan fatty acid and a cholesterol metabolite accelerate wound healing

Keywords: F6 (furan F-acid), S5 (cholesta-3,5-diene), FB (Fraction-B), Gulf catfish lipids, wound healing, histology, collagen, fibronectin, fibroblasts, leukocyte, human, rat, zebrafish

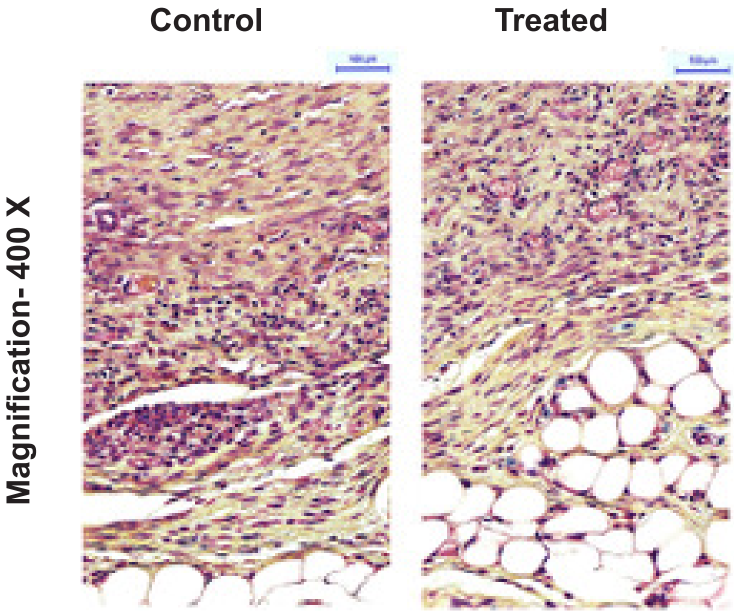

Figure 1S. FB promotes recruitment of increased number of immune cells to the wound site (400 x). Leukocytes (round cells) and fibroblasts (elongated cells with space in between containing yellow collagen).

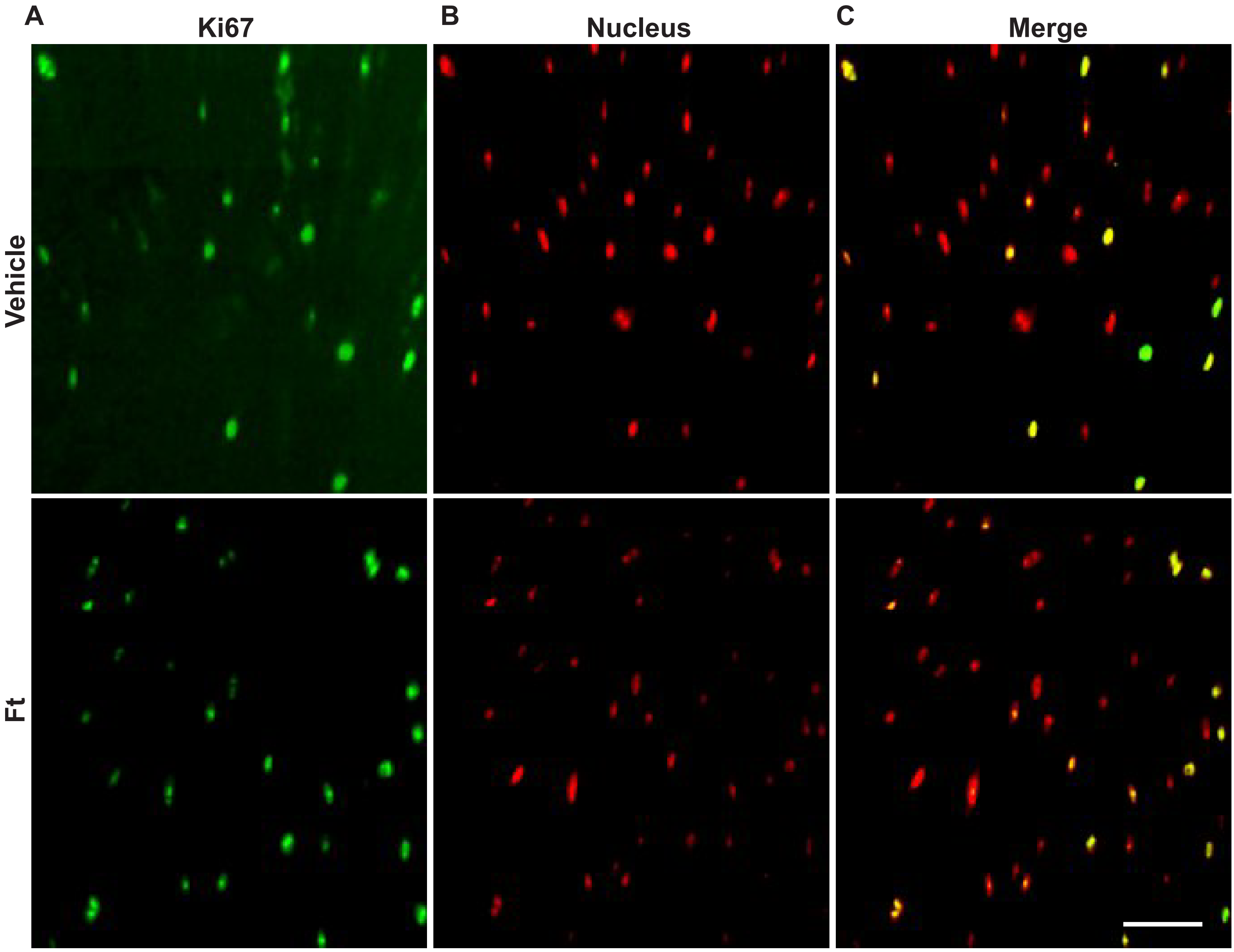

Figure 2S. Ft does not promote fibroblast proliferation. Investigation of the effects of Ft and vehicle on fibroblast proliferation measured with a cell proliferation marker, Ki67, in dermal human fibroblasts after 24 h incubation, ex vivo. **A**, green fluorescent stain, Ki67; **B**, red, propidium iodide nuclear stain; **C**, merged images.

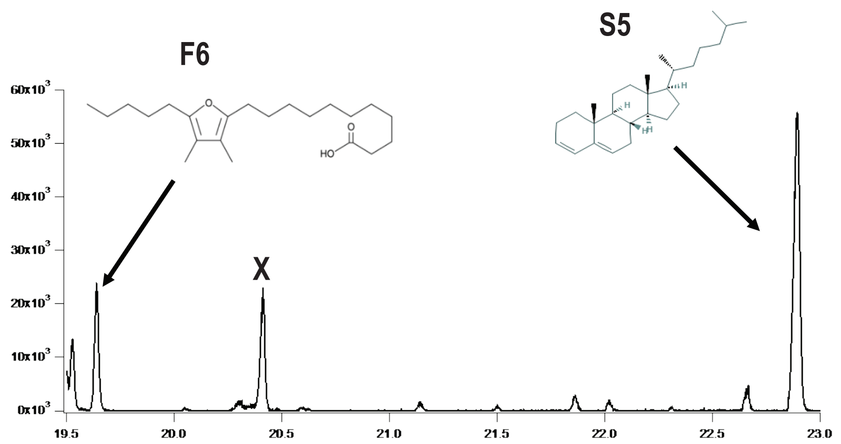

Figure 3S. GC-MS profile showing that F6 and S5 are minor components of Ft. Gas chromatogram of the Ft lipid fraction as the methyl ester derivative showing the presence of furan fatty acid (F6), and the cholesterol metabolite S5, the two compounds reported in this study. Peak X represents a non-lipid impurity.

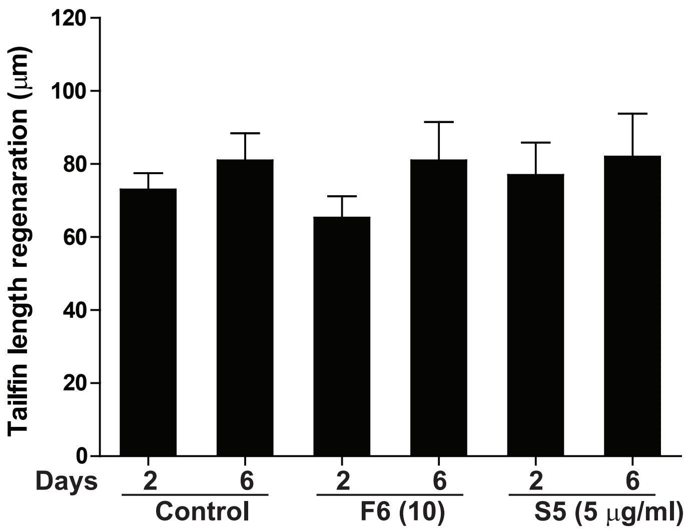

Figure 4S. F6 and S5 do not promote effective regeneration of injured tailfin in transgenic zebrafishes.
